# Similarities of P1-Like Phage Plasmids and Their Role in the Dissemination of *bla*_CTX-M-55_

**DOI:** 10.1128/spectrum.01410-22

**Published:** 2022-09-07

**Authors:** Mianzhi Wang, Li Jiang, Jingyi Wei, Heng Zhu, Junxuan Zhang, Ziyi Liu, Wenhui Zhang, Xiaolu He, Yuan Liu, Ruichao Li, Xia Xiao, Yongxue Sun, Zhenling Zeng, Zhiqiang Wang

**Affiliations:** a College of Veterinary Medicine, Yangzhou University, Yangzhou, China; b Jiangsu Co-Innovation Center for Prevention and Control of Important Animal Infectious Diseases and Zoonoses, Yangzhou, China; c Priority Academic Program Development of Jiangsu Higher Education Institutions, Yangzhou, China; d International Research Laboratory of Agriculture and Agri-Product Safety, Ministry of Education of China, Yangzhou, China; e College of Veterinary Medicine, South China Agricultural University, Guangzhou, China; f National Laboratory of Safety Evaluation (Environmental Assessment) of Veterinary Drugs, Guangzhou, China; g National Risk Assessment Laboratory for Antimicrobial Resistance of Animal Original Bacteria, Guangzhou, China; h Guangdong Laboratory for Lingnan Modern Agriculture, Guangzhou, China; Instituto de Higiene

**Keywords:** P1, phage plasmid, ESBLs, *bla*_CTX-M-55_, IncY, p0111

## Abstract

The P1-like phage plasmid (PP) has been widely used as a molecular biology tool, but its role as an active accessory cargo element is not fully understood. In this study, we provide insights into the structural features and gene content similarities of 77 P1-like PPs in the RefSeq database. We also describe a P1-like PP carrying a *bla*_CTX-M-55_ gene, JL22, which was isolated from a clinical strain of Escherichia coli from a duck farm. P1-like PPs were very similar and conserved based on gene content similarities, with only eight highly variable regions. Importantly, two kinds of replicon types, namely, IncY and p0111, were identified and can be used to specifically identify the P1-like phage. JL22 is similar to P1, acquiring an important foreign DNA fragment with two obvious features, namely, the plasmid replication gene *repA′* (p0111) replacing the gene *repA* (IncY) and a 4,200-bp fragment mobilized by IS*1380* and IS*5* and containing a *bla*_CTX-M-55_ gene and a *trpB* gene encoding tryptophan synthase (indole salvaging). The JL22 phage could be induced but had no lytic capacities. However, a lysogenic recipient and intact structure of JL22 virions were observed, showing that the extended-spectrum β-lactamase *bla*_CTX-M-55_ gene was successfully transferred. Overall, conserved genes can be a good complement to improve the identification efficiency and accuracy in future screening for P1-like PPs. Moreover, the highly conserved structures may be important for their prevalence and dissemination.

**IMPORTANCE** As a PP, P1 DNA exists as a low-copy-number plasmid and replicates autonomously with a lysogenization style. This unique mode of P1-like elements probably indicates a stable contribution to antibiotic resistance. After analyzing these elements, we show that P1-like PPs are very similar and conserved, with only eight highly variable regions. Moreover, we observed the occurrence of replicon IncY and p0111 only in the P1-like PP community, implying that these conserved regions, coupled with IncY and p0111, can be an important complement in future screening of P1-like PPs. Identification and characterization of JL22 confirmed our findings that major changes were located in variable regions, including the first detection of *bla*_CTX-M-55_ in such a mobile genetic element. This suggests that these variable regions may facilitate foreign DNA mobilization. This study features a comprehensive genetic analysis of P1-like PPs, providing new insights into the dissemination mechanisms of antibiotic resistance through P1 PPs.

## INTRODUCTION

Antimicrobials, including antibiotics, have been developed and applied in important medical procedures and have saved millions of lives since their discovery in 1920s (1). Unfortunately, antimicrobial resistance among clinically important priority pathogens is increasing and has become a serious public health problem worldwide, thereby threatening the efficacy of clinical treatments. *Enterobacteriaceae* strains resistant to third-generation cephalosporins are recognized among the critical priority pathogens by the WHO (2). The wide and inappropriate use of antimicrobials has exacerbated the development of resistance (3). Acquired resistance to cephalosporins is mediated principally by extended-spectrum β-lactamases (ESBLs), the most prevalent of which are the ESBLs of the CTX-M, TEM, and SHV families (4). Evidence has shown that plasmid-located *bla*_CTX-M_-, *bla*_TEM_-, *bla*_SHV_-, and *bla*_OXA_-type genes are ubiquitous (5).

Antimicrobial resistance genes (ARGs) can be mobilized by horizontal gene transfer (HGT) from one bacterium into another recipient in three ways, i.e., conjugation (cell to cell), transduction (phage mediated), or transformation (direct absorption of naked DNA from dead cells) (6). However, the dissemination of ARGs mediated by transduction of phages (bacteriophages) has been underestimated. Phages are the most abundant (~10^30^ phages and ~10^25^ infections/s) biological entities on Earth (7, 8). The host range of transducing phages can also be broad, and the transduction of ARGs is likely to be common worldwide, allowing them to infect different bacterial species and to spread ARGs in natural environments (9).

Phages can promote transfer of ARGs, among which ESBL family genes are widely found, via transduction. Colomer-Lluch and colleagues revealed the existence of two ESBL-encoding genes (*bla*_TEM_ and *bla*_CTX-M_) in phage DNA from animal feces in slaughterhouse and water samples from sewage and a river (10–13). ESBL-coding genes have also been recovered worldwide from different samples, i.e., *bla*_TEM_, *bla*_CTX-M_, and *bla*_SHV_ in hospital effluents (14); *bla*_OXA-2_ in a chicken farm and its surrounding water, as well as soils and sediments, in India (15); *bla*_TEM_ and *bla*_CTX-M-1_ in three large-scale pig farms (16); *bla*_TEM_, *bla*_CTX-M-1_, *bla*_CTX-M-9_, *bla*_OXA-48_, *bla*_TEM_, *bla*_CTX-M-1_, *bla*_CTX-M-9_, *bla*_OXA-48_, and *bla*_VIM_ in fresh-cut vegetables and soils (17); *bla*_TEM_, *bla*_CTX-M_, *bla*_PSE_, and *bla*_CMY-2_ in the Yakima River (18); and *bla*_CTX-M_ in 30 different samples of chicken feces (19).

Although studies have explored the abundance and distribution of ESBL genes from diverse environmental phage metagenomic samples (20), identification of ESBL-carrying phages and the mechanism of specific dissemination remain largely unclear. A previous study reported that *bla*_CTX-M-10_ linked to phage-related elements can be transferred from the chromosome to a plasmid via phage transduction (21). Also, a study showed that staphylococcal phages and pathogenicity islands promoted plasmid evolution (22). These results imply a possible relationship between plasmids and phages (23). In the following years, several P1-like phages carrying ESBL family genes were successively identified and characterized; these include phage RCS47 harboring *bla*_SHV-2_ (24) and phage SJ46 harboring *bla*_CTX-M-27_ (25). On the other hand, evidence indicates that phage plasmids (PPs) have an extremely wide distribution in bacterial populations (26) and, in that regard, P1-like PPs are also widely present in *Enterobacteriaceae* strains (24). Phage P1 (GenBank accession number NC_005856), a temperate phage, induces the lytic or lysogenic pathway upon infecting Escherichia coli and several other enteric bacteria. Usually, P1 DNA exists as a low-copy-number plasmid and replicates autonomously with a lysogenization style independent of multiplicity of infection (MOI) (27). This unique characteristic ensures stable lysogenic ability even at low concentrations of P1 phage and may contribute to facilitating the dissemination of ESBL-family genes.

In this study, in order to further explore the similarities of P1-like elements, 77 P1-like PP genome sequences were selected to gain insights into the abundance and structural features of P1-like phages, as well as similarities in their gene content among different groups, which will provide a new understanding of their contribution in spreading antimicrobial resistance. Furthermore, a P1-like phage carrying *bla*_CTX-M-55_, JL22, which was isolated from an E. coli strain from a duck, was identified and characterized to clarify its role in the dissemination of *bla*_CTX-M-55_.

## RESULTS

### Bacterial identification.

Antimicrobial susceptibility testing showed that 67 of 103 E. coli isolates displayed ceftiofur MICs of ≥256 μg/mL, suggesting the presence of a putative ESBL. These isolates were further analyzed for P1-like PP identification.

### Genetic composition of P1-like PPs.

**FIG 1:**
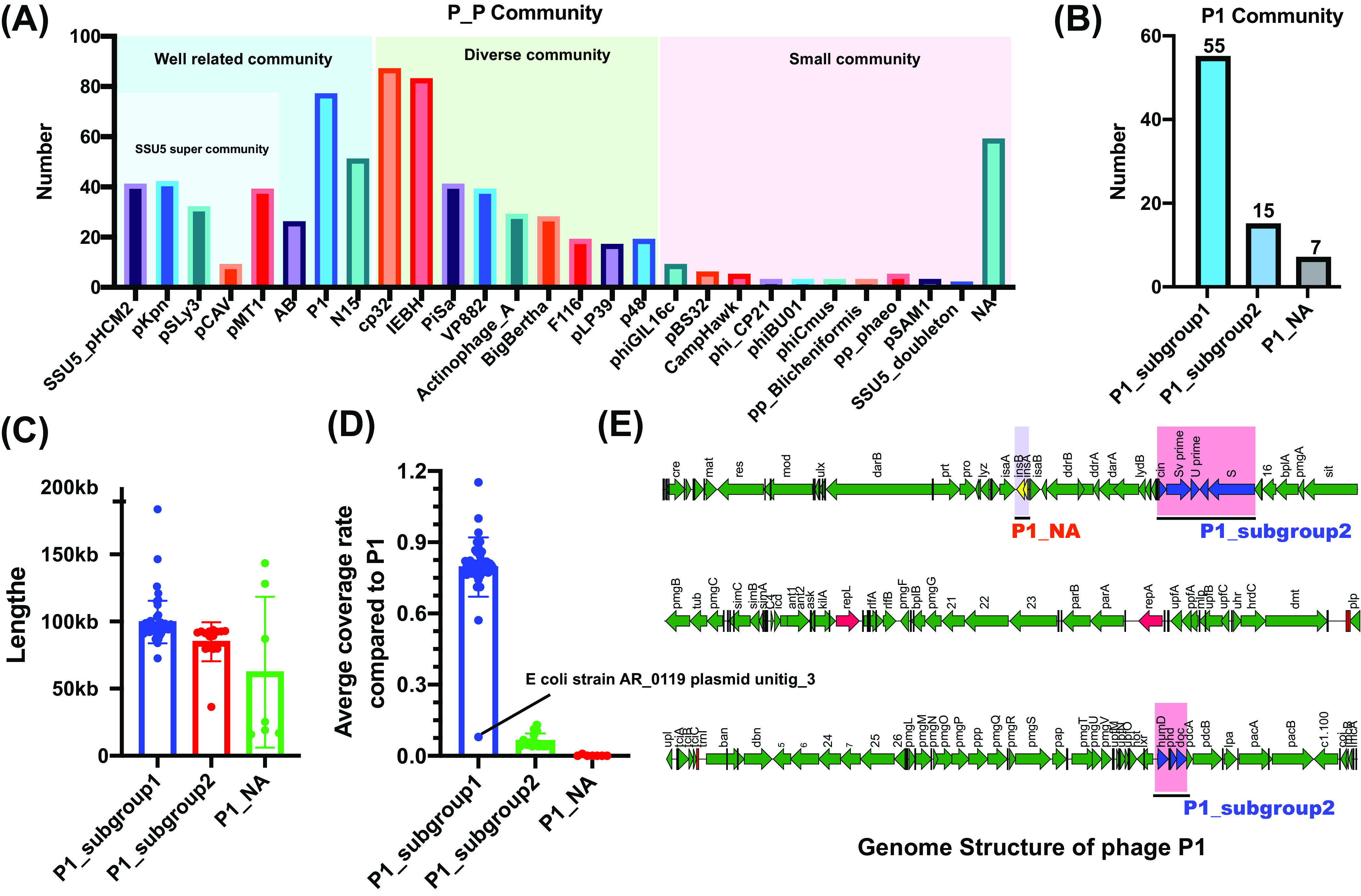
Genetic composition of 77 P1-like PPs. (A) Distribution and classification of 780 PPs. (B) Distribution and classification of the three P1-like PP community subgroups. (C) Length distribution of the three P1-like PP community subgroups. (D) Average coverage rate of 54 representative P1-like PPs, compared with P1. (E) Shared genes of P1 subgroup 2 and the P1 NA subgroup, compared with P1. Colored rectangles represent shared genes, with light purple and light red representing shared genes (*insA-insB*) of the P1 NA subgroup, compared with P1, and shared genes (*cin-Sv′-U’-U-S* and *humD-phd-doc*) of P1 subgroup 2, compared with P1, respectively.

According to a recent study (26), 780 PPs were divided into three communities, namely, a well-related community, a diverse community, and a small community. In the well-related community, P1-like PPs were the most frequent, accounting for 9.87% of all PPs (77/780 PPs), and most belonged to subgroup 1 (55/77 PPs) (Fig. 1A and B). The BLAST results between these 77 phages and P1 showed significant differences among P1-like subgroup 1, P1-like subgroup 2, and the P1-like not assigned (NA) group. P1-like subgroup 1 matched about 80.89% of the length of P1 (except for E. coli strain AR_0119 plasmid unitig 3 [similar to group 2]), while the value for group 2 was only 6.39%, and the only sequence that could be retrieved for the NA group (Shigella flexneri 1a strain 0228 plasmid) matched 0.81% of the length of P1 (Fig. 1D); this suggests that the remaining 54 p1-like PPs in P1-like subgroup 1 are more representative of the P1 community. Although these three groups have a low level of similarity in genome length (Fig. 1C), several shared genes were identified (Fig. 1E), such as the *cin-Sv-U-S* cluster (encoding site-specific recombinase and tail fiber) and the *humD-phd-doc* cluster (encoding SOS response protein and a toxin-antitoxin [TA] system protein) in subgroup 2 and the *insA-insB* cluster (IS*1*) in the NA group.

### P1-like PP sequences display highly variable and conserved properties.

**FIG 2:**
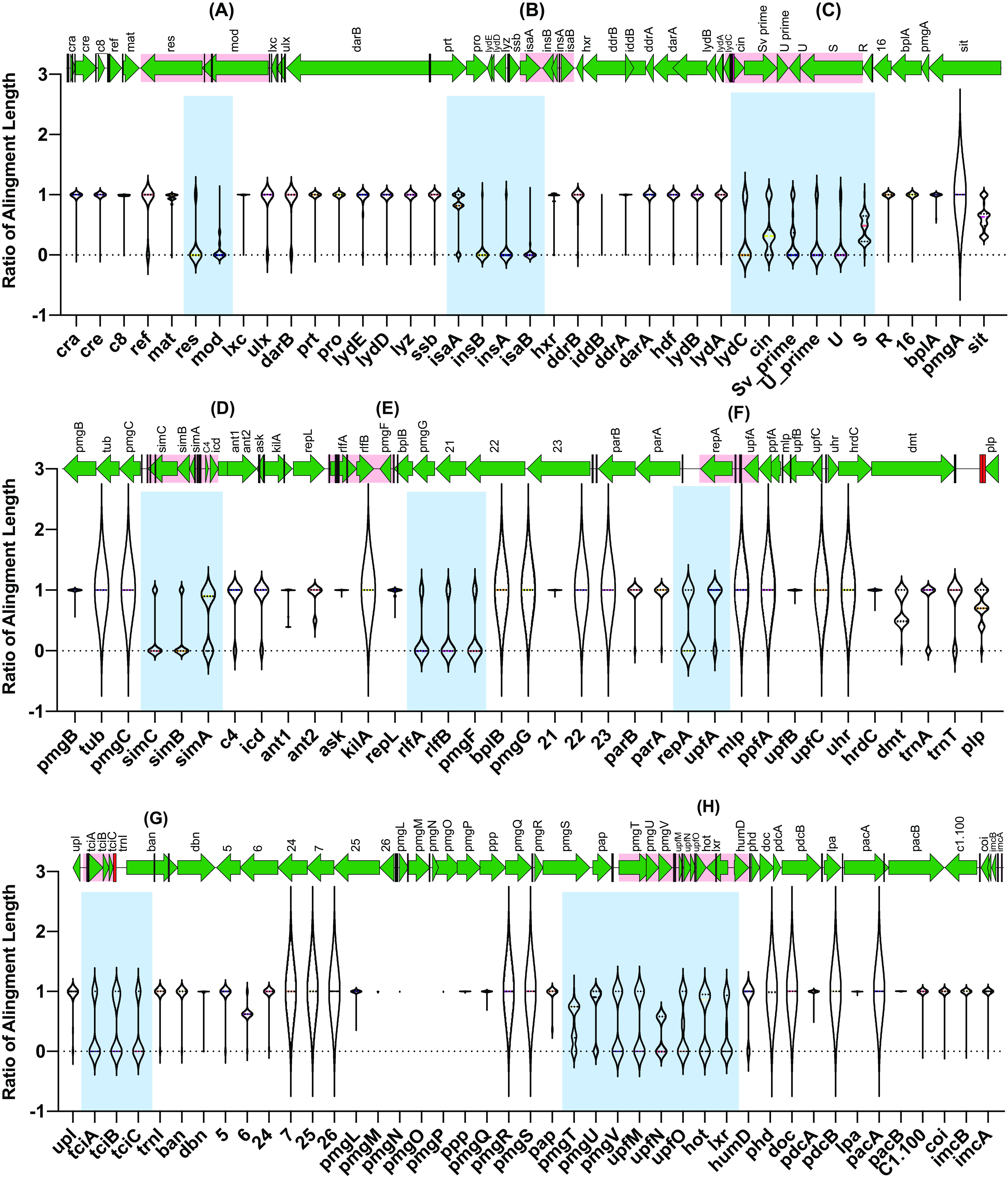
Gene content similarities between all 117 genes of the P1 genome and 54 representative P1-like PPs. The light blue shaded areas represent the eight highly variable regions (regions A to H). (A) *res-mod*. (B) *isaA*, *insB*, *insA*, and *isaB*. (C) *lydC*, *cin*, *Sv′*, *U′*, *U*, and *S*. (D) *simC*, *simB*, and *simA*. (E) *rlfA*, *rlfB*, and *pmgF*. (F) *repA* and *upfA*. (G) *tciA*, *tciB*, and *tciC*. (H) *pmgT*, *pmgU*, *pmgV*, *upfM*, *upfN*, *upfO*, *hot*, *lxr*, and *humD*. The light red shaded areas represent the corresponding positions of the eight highly variable regions in P1 phage.

Analysis of the remaining 54 P1-like PPs and the alignment results among all 117 genes of the P1 genome and the 54 P1-like PPs showed that the 54 P1-like PPs are very similar and conserved. However, eight highly variable regions outside the conserved regions were also detected, as shown in Fig. 2, i.e., region 1 (*res-mod* [restriction-modification protein]) (Fig. 2A), region 2 (*isaA*, *insB*, *insA*, and *isaB* [IS*1*-associated genes]) (Fig. 2B), region 3 (*lydC*, *cin*, *Sv′*, *U′*, *U*, and *S* [encoding holing and tail-fiber related protein]) (Fig. 2C), region 4 (*simC*, *simB*, and *simA* [superimmunity-linked function]) (Fig. 2D), region 5 (*rlfA*, *rlfB*, and *pmgF* [possibly replication-linked function]) (Fig. 2E), region 6 (*repA* and *upfA* [plasmid replication]) (Fig. 2F), region 7 (*tciA*, *tciB*, and *tciC* [tellurite or colicin resistance or inhibition of cell division]) (Fig. 2G), and region 8 (*pmgT*, *pmgU*, *pmgV*, *upfM*, *upfN*, *upfO*, *hot*, *lxr*, and *humD* [putative morphogenetic function or SOS putative morphogenetic function]) (Fig. 2H).

Among the conserved genetic regions, 21 extremely conserved genes (*pmgA*, *tub*, *pmgC*, *kilA*, *bplB*, *pmgG*, *gp22*, *gp23*, *mlp*, *ppfA*, *upfC*, *uhr*, *gp7*, *gp25*, *gp26*, *pmgR*, *pmgS*, *phd*, *doc*, *pdcB*, and *pacA*) were also observed to be evenly distributed in different sites of the P1 genome. The length of these genes matched 100% of the corresponding P1 gene. The other genes in the conserved region also showed high levels of stability regardless of harboring several base substitutions on deletions, including P1 lytic replication gene *repL*. The detection of these genes, instead of *repL*, could improve the future identification of P1-like subgroup 1 PPs. It is worth noting that genes *phd* and *doc* were excluded because they are also the core genetic part of P1-like subgroup 1.

### P1-like PPs carrying the *bla*_CTX-M-55_ gene.

Based on the conserved gene repertoire, nine genes, i.e., *gp22*, *gp23*, *gp25*, *pmgS*, *pdcB*, *ppp*, *hdf*, *pacA*, and *repL*, were selected to identify potential P1-like PPs from the 67 E. coli strains carrying putatively ESBL-coding genes. Among them, 4 isolates yielded positive PCR results. However, combined Illumina HiSeq 2500 and Oxford Nanopore Technologies MinION sequencing showed that only one E. coli strain, named strain 22, carried the ESBL gene-bearing P1-like PP JL22. The complete sequence of JL22 is 99,605 bp long and harbors 124 open reading frames (ORFs), accounting for 90% of its genome. For better visualization of JL22, the genome was represented as a circle with the site-specific recombination site *lox* assigned to the zero position, because P1 and similar phages lysogenize their hosts as autonomous plasmid-like elements. The GC content of JL22 is 47.48%. We found that 88.2% of JL22 showed 95.7% nucleotide identity to P1 phage.

**FIG 3:**
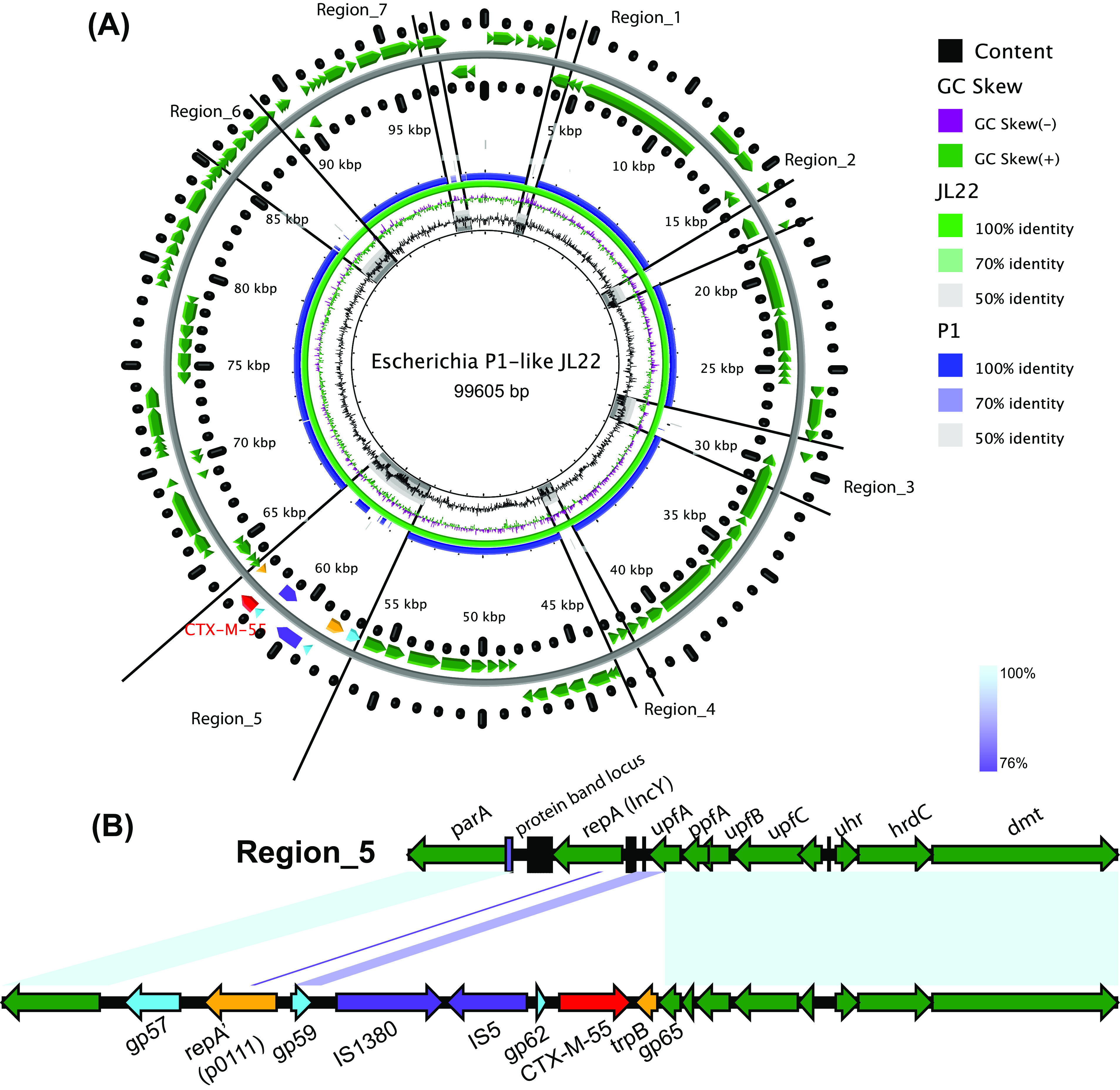
Circular representation of the organization of the phage JL22 genome and representation of the synteny between phage JL22 and P1 (GenBank accession number NC_005856). (A) Distribution of the seven regions, showing differences in relation to P1. (B) Genetic environment of *bla*_CTX-M-55_ gene-containing region 5.

**FIG 4:**
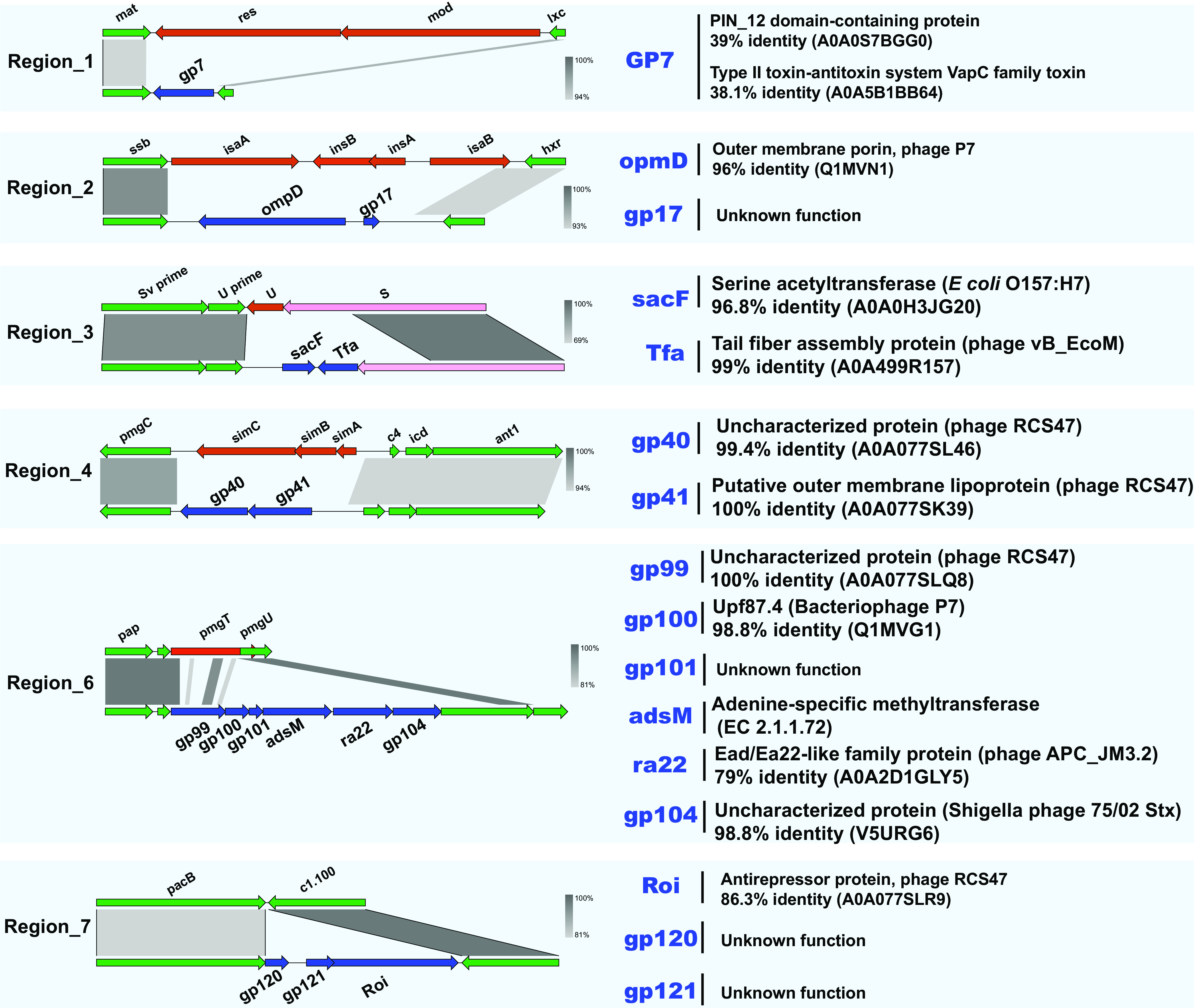
Genetic environment of the other six regions in JL22. (Left) Synteny between phage JL22 and P1. (Right) Gene function within the replaced region.

**FIG 5:**
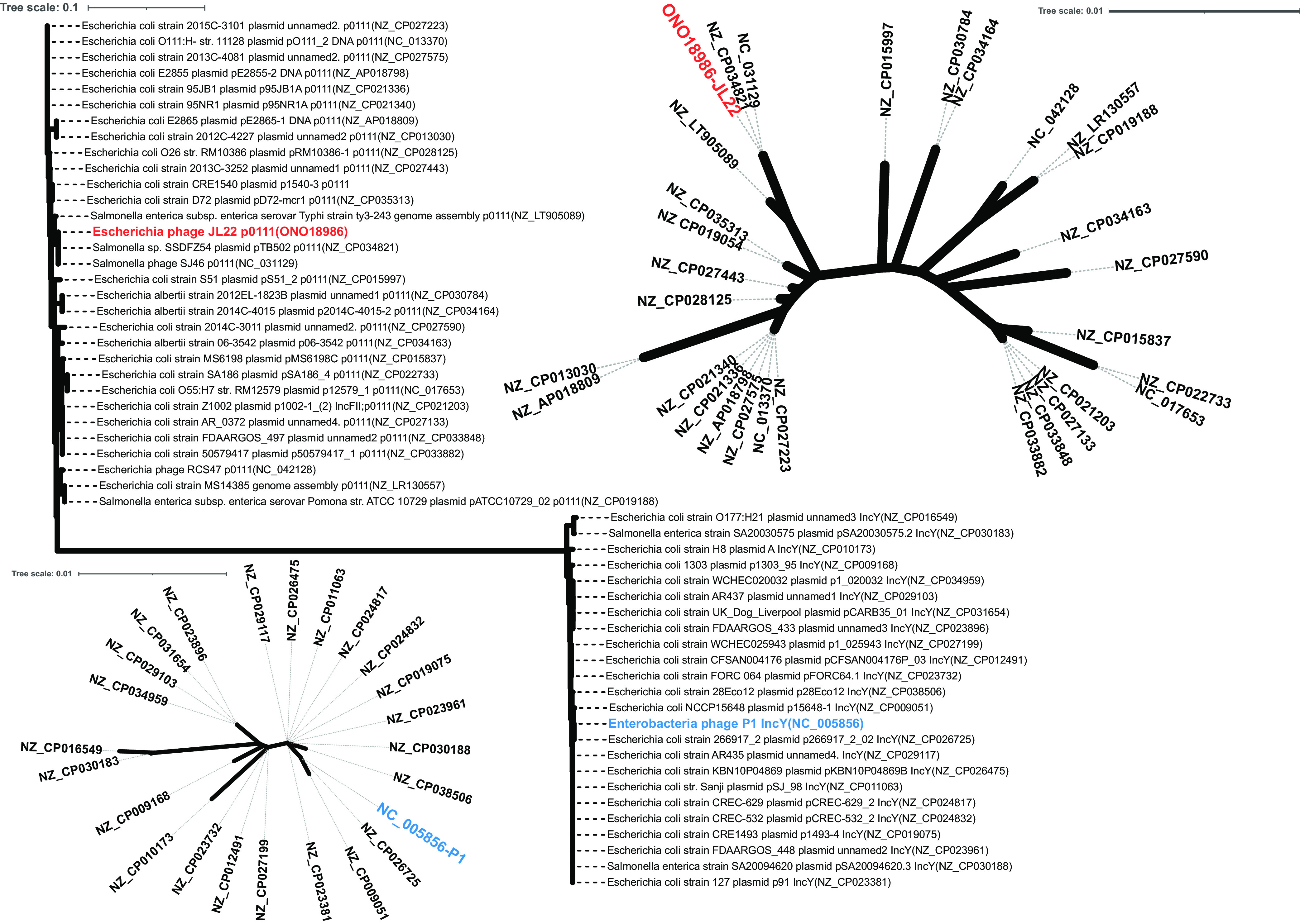
Phylogenetic analysis of plasmid replication genes (IncY and p0111) based on sequences of the 54 representative P1-like PPs. The upper right area represents the phylogeny of p0111, and the lower left area represents the phylogeny of IncY.

JL22 has seven regions showing major differences in relation to P1 (Fig. 3A). Except for region 5, the other six regions spanned no more than 5 kb, and all were located in the highly variable regions of the P1-like PP sequence (Fig. 4). Region 5 shows two main features. First, the plasmid replication gene *repA′* (p0111 plasmid incompatibility group) and an unknown protein-encoding gene gp57 replacing the gene *repA* (IncY plasmid incompatibility group). Two plasmid incompatibility groups were identified among the 55 P1-like subgroup 1 PPs (including JL22), i.e., IncY (31/55 PPs) and p0111 (24/55 PPs). The phylogenetic analysis results for the two plasmid replication genes showed that IncY and p01111 had significantly evolutionary differences, whereas each plasmid replication gene sequence highly maintained conservation (Fig. 5; also see Table S1 in the supplemental material). Importantly, the distribution rates of the two replication genes were almost the same. Second, a 4,200-bp DNA sequence was inserted at nucleotide position 63151 (ORF at positions 62950 to 63342), interrupting a 392-bp gene *upfA* of unknown function, downstream from a membrane lipoprotein precursor-encoding gene, *mlp*. This segment contains a *bla*_CTX-M-55_ gene surrounded upstream by the insertion sequences IS*1380* and IS*5* and downstream by the *trpB* gene encoding the tryptophan synthase (indole salvaging). No other ARGs were detected in JL22 (Fig. 3B and 4).

### Absence of lytic capacity and lysogenization of phage JL22.

As a PP, *bla*_CTX-M-55_-bearing JL22 might facilitate its horizontal dissemination through lysis-lysogeny pathways. We then investigated whether the JL22 phage (i) could be induced, (ii) had lytic capacities, and (iii) could transfer *bla*_CTX-M-55_.

Following mitomycin C induction, we obtained a JL22 suspension containing *bla*_CTX-M-55_ from the natural isolate 22, which also yielded positive PCR results for *gp22*, *gp23*, *gp25*, *pmgS*, *pdcB*, *ppp*, *hdf*, *pacA*, and *repL*. The PCR assay aimed at detecting the 16S rRNA gene yielded negative results, ruling out any possible contamination with extracellular bacterial DNA.

The lytic capacity of JL22 was assessed by spotting the suspensions onto agar overlays of E. coli strain MG1655, which is widely used for P1 assays. After 24 h of incubation at 37°C, the concentrated suspensions of JL22 did not induce a plaque.

**FIG 6:**
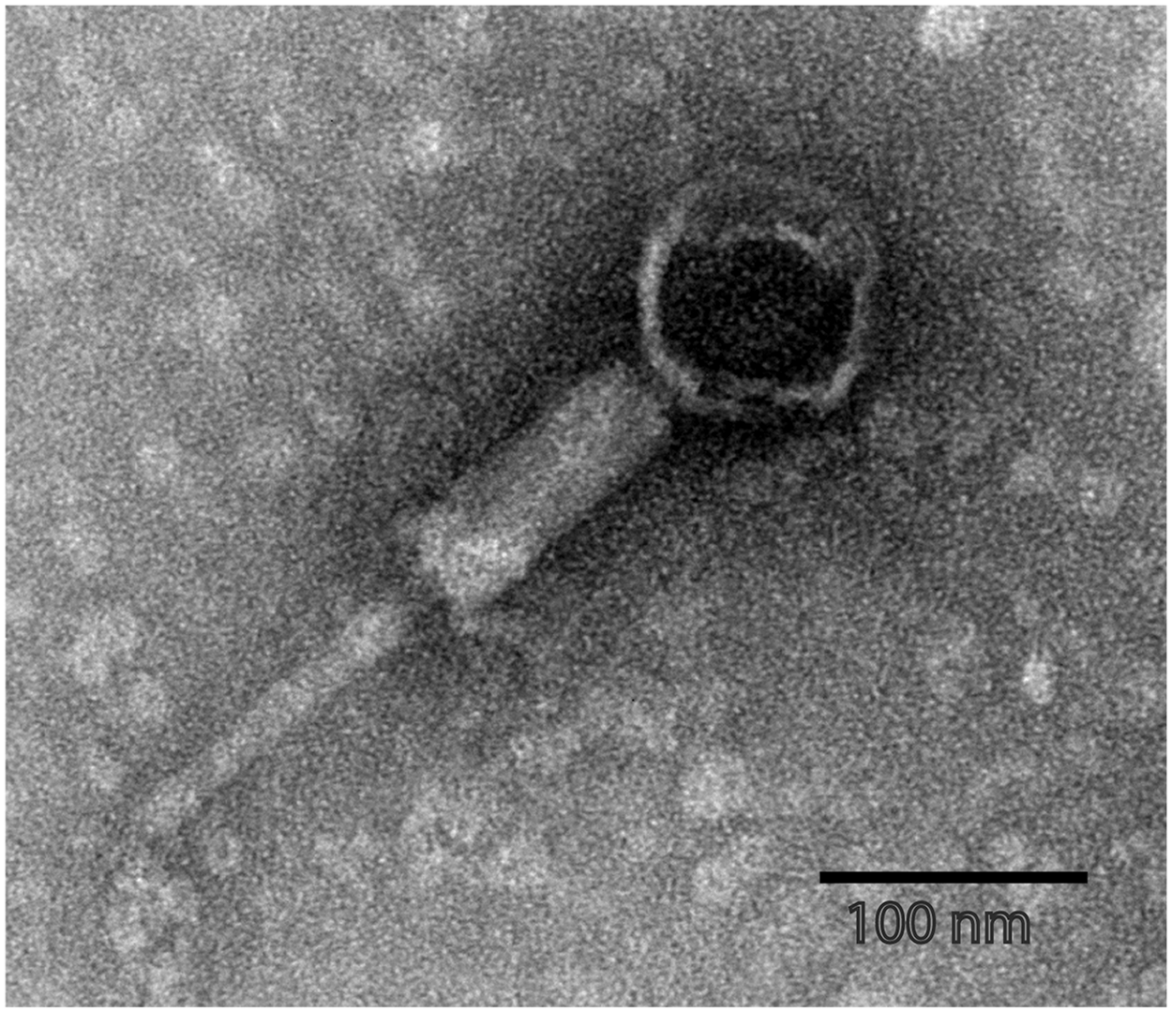
Electron microscopy of phage JL22, showing the presence of an icosahedral DNA-containing head of approximately 75 nm and a structure resembling the tail tube.

We also assessed the ability of JL22 to lysogenize E. coli strain MG1655 and thus to transfer *bla*_CTX-M-55_. Using the lysogenization procedure described above, we obtained colonies resistant to third-generation cephalosporins, and PCR assays indicated the presence of nine JL22 genes. To better understand the morphology of JL22, we prepared phage suspensions from E. coli strain 22 and examined them by electron microscopy. They showed the presence of icosahedral DNA-containing heads of about 75 nm and structures resembling tail tubes (Fig. 6).

## DISCUSSION

The presence and distribution of PPs have been fairly well investigated using genomic analyses (26, 28). However, although phage P1 was discovered over 50 years (29, 30), research efforts have mainly focused on its properties as a molecular biology tool (31), and its role as an active element of the accessory cargo genome with dual properties of plasmid and phage has not been fully understood. Therefore, in-depth insights into the abundance and structural features of P1-like elements, as well as their gene content similarities among different groups, will provide a new understanding of their contribution in spreading antimicrobial resistance. In this study, we determined the underlying relationship and genetic composition of 77 P1-like PPs and analyzed the relationship of the three subgroups. A P1-like PP carrying *bla*_CTX-M-55_, JL22, which was isolated from E. coli in a duck farm in China, was identified and characterized.

In a recent study, Pfeifer et al. identified 780 PPs screened from 11,827 plasmids and 2,502 phages retrieved from the NCBI nonredundant RefSeq database, accounting for ~7% of the sequenced plasmids and ~5% of the sequenced phages (26). Further comparison of the 77 P1-like PPs to the reference genome (phage P1) indicated that three regions (*cin-Sv′-U′-U-S*, *humD-phd-doc*, and *insA-insB*) were similar to the P1 genes, and they are also located in highly variable regions of P1.

Evidence indicates that *U* (*U* and *U’*), *S* (*Sv* and *Sv′*), and R operons determine host specificity (32), and their high variability might imply a wider host spectrum. For *humD-phd-doc* clusters, the *humD* gene is a LexA-regulated gene of the SOS response (33), and the *phd*-*doc* operon functions as a TA system (34). For the *ins* (*insA-insB*) operon, the two adjacent genes are required for IS*1* transposition and IS*1*-mediated plasmid cointegration (35). Evidence indicates that IS*1* can mediate the transfer of ARGs (36), and the region upstream of *insA* seems to be an integration hot spot. Billard-Pomares et al. (24) reported that the presence of *bla*_SHV-2_ is involved in recombination at this position. In summary, these results suggested that the three matched gene regions (*cin-Sv′-U’-U-S*, *humD-phd-doc*, and *insA-insB*) might play a key role in P1 community maintenance and stabilization. Nevertheless, it was still worth noting that the highly conserved genome of P1-like subgroup 1 is more representative of the P1 community, and the eight highly variable regions probably are the hot spot in ARGs mobilization.

Evidence shows that the variable regions of P1-like PPs were associated with the low G+C content, including *res*-*mod*, *isaA*-*IS1*-*isaB*, *simABC*, *rlfAB*, and *hox-lxr* regions. Also, inspection of other P1-like PPs described in the literature showed that these regions also tend to be shared among different P1-like elements isolated from different hosts (37), suggesting that the GC content probably has an important effect on foreign gene mobilization regardless of the host spectrum. Surprisingly, the *tciABC* operon does not exhibit low G+C content; furthermore, nearly one-half of the tested P1 subgroup 1 PPs lost this operon sequence. The predicted products of P1 *tciA* showed that they have significant similarities to the gene *terB*. The *terB* gene is an internal gene of multigenic *ter* operons, which have been implicated in tellurite resistance, phage inhibition, and pathogenicity; they are usually located on prophage-like elements of IncHI2 plasmids or the chromosome (38, 39). Whether the high variability of *tciABC* operons is related to phage inhibition remains unclear.

Apart from the eight highly variable genetic regions, 21 extremely conserved genes with 100% identity to genes in the P1 genome (*pmgA*, *tub*, *pmgC*, *kilA*, *bplB*, *pmgG*, *gp22*, *gp23*, *mlp*, *ppfA*, *upfC*, *uhr*, *gp7*, *gp25*, *gp26*, *pmgR*, *pmgS*, *phd*, *doc*, *pdcB*, and *pacA*) were found, indicating that these genes may be indispensable for P1-like PPs, such as the *phd*-*doc* operon encoding a TA system. For the other genes, most of which were identified as unknown or coding for putative proteins, additional information is needed to validate the role of these putative functional genes in the maintenance and stabilization of P1-like PPs. Moreover, the identification of *repL* among the 54 P1-like PPs showed that *repL* was located in the conserved region although it did not exhibit 100% identity to P1 *repL* (identity ranging from 97.3% to 99.41%), and the result indirectly confirmed the previous observations that the lytic replication gene *repL* could be used as a specific PCR target to detect the presence of P1-like phages.

On the other hand, our results showed that there are two different types of plasmid replication genes in P1-like PPs, belonging to IncY and p0111. Moreover, phylogenetic analysis showed that the two genes had a significant evolutionary difference and intergroup gene sequences remained highly conserved, indicating that the two replicon types can also be used to specifically identify the P1-like PPs (40–43). With the ease and accessibility of whole-genome sequencing, more plasmid sequences can be easily obtained. Also, our results can provide a good supplement to accurately and efficiently screen P1-like PPs with searches for the replicon gene (IncY or p0111) and PCR verification of the nine highly conserved genes.

Although ESBL genes, such as *bla*_CTX-M-27_ (25, 44), *bla*_CTX-M-15_ (37, 45, 46), *bla*_KPC-2_ (47), and *bla*_SHV-2_ (24), have been found in different P1-like PPs, the present study is the first to report *bla*_CTX-M-55_-positive P1-like PPs. According to recent studies, the gene *bla*_CTX-M-55_ was found to be mainly located on IncFII-type plasmids and also was sporadically detected in IncN, IncI1, IncFIC, IncFIB, IncHI2, and IncI2 (48–50) within a homologous region of IS*26*-*bla*_TEM_-*orf477*-*bla*_CTX-M-55_-IS*Ecp1*-IS*26*. Hence, IS*Ecp1*-IS*26* was probably involved in the spread of *bla*_CTX-M-55_ (51), whereas no IS*26-* or IS*Ecp1*-related insertion sequence was detected in JL22, except for the insertion sequences *IS*1380 and *IS*5. A study stated that *bla*_CTX-M-32_ was located in the chromosome, upstream of IS*5*-like and IS*1380*-like sequences, in a bovine cecal sample-derived E. coli strain; also, a *bla*_CTX-M-2_ gene was in the p0111 plasmid, flanked by *orf3*/*qacEΔ1* and IS*91*/IS*CR1* (52). Considering the cooccurrence of IS*5*-like and IS*1380*-like sequences and p0111 plasmid in a single strain, we then speculated that the *bla*_CTX-M-55_ location in JL22, a p0111 plasmid, might have a closer relationship with chromosomal sequences or even be derived from chromosomes.

As a PP, phage JL22 might spread *bla*_CTX-M-55_ horizontally through lysis-lysogeny pathways (53). Following mitomycin C induction, JL22 was successfully induced but had no lytic capacities, as detected by spotting assays. Despite the lack of lytic ability, JL22 had the ability to lysogenize MG1655 and thus to transfer *bla*_CTX-M-55_. Electron microscopy of JL22 showed the presence of icosahedral DNA-containing heads of about 75 nm and neck, tail, and baseplate structures, implying that JL22 retains its intact structure, compared with the original structure of P1 (54, 55). Before lysogenization assays were performed, the analysis of the JL22 genome revealed that two structural gene regions were replaced by other functional protein genes, i.e., region 3 and region 6.

For region 3, the *U* operon and part of the *S* operon were replaced by *sacF* and *Tfa* genes, respectively. As stated previously, *U*, *S*, and *R* operons are involved in tail fiber production and host specificity; in this regard, this is probably a substitution by a homologous gene since gene *tfa* also encoded a tail fiber assembly protein. On the other hand, although JL22 and P1 preserved the lysogenic ability, whether they suffered any changes in their host range remain unclear. Interestingly, among the changed regions, a gene encoding serine acetyltransferase was identified. Serine acetyltransferase, an enzyme involved in catalyzing the first step of cysteine biosynthesis, is essential for the survival of persistent microbes and therefore is usually recognized as a target for identifying potential inhibitors (56). However, the reason for the presence of a serine acetyltransferase gene in the P1-like PP JL22 is still unknown. In region 6, the *pmgT* gene was replaced by a series of gene clusters, i.e., *gp99*-*pg100*-*gp101*-*adsM*-*ra22*-*gp104*, most of which are genes encoding proteins of unknown function. While the replacement of *pmgT* did not influence the lysogenic ability, further studies are still required to explore the potential roles of these genes.

## MATERIALS AND METHODS

### Bacterial isolate and antimicrobial susceptibility.

Susceptibility to ceftiofur was assessed for 103 E. coli isolates that had been recovered from healthy ducks in 2020 in China, to screen for putative ESBL producers. Antimicrobial susceptibility testing was conducted by the broth microdilution method in accordance with the standards and guidelines described by the Clinical and Laboratory Standards Institute (CLSI) guidelines (57). Escherichia coli ATCC 25922 was used as the quality control.

### Genetic composition of P1-like PPs and target gene selection.

Seventy-seven P1-like PPs (see Table S1 in the supplemental material) were obtained from the NCBI nonredundant RefSeq database (26). These genome sequences were then aligned with all 117 genes of the P1 reference sequence by using local BLAST+. Only those genes with E values of ≤1*e* − 5 and identity of ≥80% were taken into consideration and normalized against their corresponding P1 reference gene (58). P1-like PPs were efficiently and specifically screened from previously isolated bacteria. Based on the genetic composition analyzed in the previous item, nine genes were selected for further PCR-based assays, namely, *repL* (phage lytic replication gene), *pmgS* (putative morphogenetic function), *ppp* (serine/threonine protein phosphatase gene), *pacA* (phage DNA-packaging gene), and five other genes of unknown function (*pdcB*, *gp22*, *gp23*, *gp25*, and *hdf*). The PCR procedure consisted of 35 cycles of denaturation at 94°C for 30 s, annealing for 30 s, and extension at 72°C for 45 s, followed by an additional 10 min of extension at 72°C. Annealing temperatures are listed in Table S2 in the supplemental material.

### Genome extraction.

Genomic DNA was extracted using the TIANamp bacterial DNA kit (TianGen, Beijing, China), following the manufacturer’s instructions. Putative PPs in strain 22 were also extracted with a plasmid midikit (Qiagen, Germany) following the manufacturer's instructions. The genomic DNA was first subjected to short-read sequencing (2 × 150 bp) with the Illumina HiSeq 2500 platform. Genomic and plasmid DNA were then sequenced with the Oxford Nanopore Technologies MinION long-read platform with the RBK004 barcoding library preparation kit and MinION R9.4.1 flow cells to obtain the complete sequences, as described previously (59).

### High-throughput sequencing and bioinformatic analysis of PP JL22.

Genomic DNA short-read Illumina and long-read Nanopore data were used to perform *de novo* assembly with the hybrid strategy as described previously (60). Short-read Illumina raw sequences were assembled using SPAdes (61). Multilocus sequence typing (MLST) of strains was performed using the MLST tool (62, 63). The draft genomes were annotated using Prokka software (64). The Flye long-read assembly tool was used to perform *de novo* assembly of Nanopore long-read MinION sequences of PPs and genomic DNA (65). The draft assembly of *bla*_CTX-M-55_-bearing P1-like PP was analyzed using the BLASTn program against the nonredundant database. High-quality complete genome sequences were annotated using RAST (http://rast.nmpdr.org) automatically and manually. Plasmid replicons, insertion sequences, and antimicrobial resistance determinants were determined using online tools (https://cge.cbs.dtu.dk/services). BRIG, CGView, and Easyfig were used to generate figures for the genetic comparison (66–68).

### Induction of PP JL22.

E. coli strain 22 was cultured to the exponential growth phase at 37°C in LB. The bacterial suspension was treated with 2.0 μM mitomycin C and incubated for 2 h at 37°C with shaking. The suspension was then centrifuged at 3,000 × *g* for 15 min to remove bacterial cell debris and filtered using a 0.22-μm Millex-GP filter (Millipore). The filtrate from the previous step was concentrated using a 100-kDa Amicon Ultra centrifugal filter unit (Millipore) to a final volume of about 1 mL. The phage suspensions obtained were stored at 4°C. The suspension containing phage JL22 was also checked by PCR for the five target genes to confirm the successful induction of JL22.

### Lytic capacity of PP JL22.

The lytic capacity of JL22 was determined as described previously (24), by using E. coli strain MG1655. Exponentially grown cells were uniformly distributed in semisolid LB agar medium and overlaid on top of LB agar; 10-μL aliquots of the pure suspensions containing phage JL22 were then spotted on the surface of the plate. After overnight incubation at 37°C, the occurrence of plaques on the plate was considered to indicate the presence of a lytic phage, and the lack of the latter indicated possible lysogenization. The suspension was also treated with trypsin for 20 min (250 μg/mL) at 30°C to rule out the occurrence of colicins affecting strain MG1655.

### Lysogenization assay of PP JL22.

The phage lysogenization assay was carried out as described by Goh et al., with minor modifications (69). Briefly, an overnight culture of the recipient strain (rifampicin-resistant E. coli C600) was mixed with JL22 suspension to achieve an MOI of 10, incubated for 1 h at 37°C, and then centrifuged at 14,000 × *g* for 30 s. The supernatant was removed, and the cells were washed in 1 mL of LB broth. Washing was repeated twice, and the cells were resuspended in 150 μL of LB broth. About 150 μL of the cells was plated on three LB agar plates supplemented with ceftiofur (256 μg/mL) and rifampicin (200 μg/mL), and the plates were incubated for 48 to 72 h at 37°C. Putative lysogenic colonies were checked for the presence of the nine target P1 genes by PCR. The recipient bacterial concentrations and JL22 concentrations were standardized to 1 × 10^7^ CFU/mL and 1 × 10^8^ PFU/mL, respectively. A control containing only bacteria and phage buffer was included in each experiment.

### Transmission electron microscopy.

Electron micrographs of purified JL22 phage particles were obtained as described below. High-titer phage stocks were concentrated 10-fold by using 100-kDa Amicon Ultra centrifugal filter units, and 15 μL of phage concentrate was dropped on carbon-coated Formvar-covered grids for 15 min. The drops were then blotted, and the samples were stained with 2% (wt/vol) phosphotungstic acid (pH 7.0) and air dried. The phages were examined with a FEI transmission electron microscope (Thermo Fisher Scientific, Hillsboro, OR, USA) at an acceleration voltage of 80 kV (70).

### Data availability.

The *bla*_CTX-M-55_-bearing JL22 PP generated in this study was deposited in the NCBI database. The complete nucleotide sequence of JL22 was deposited in GenBank under the accession number ON018986.
