# Supplementary material for: Similarities of P1-Like Phage Plasmids and Their Role in the Dissemination of blaCTX-M-55
Source: Microbiol Spectr. 2022 Sep 7;10(5):e01410-22. doi: 10.1128/spectrum.01410-22 (PMC9603915; doi:10.1128/spectrum.01410-22)
Supplement: Supplemental file 1 — Table S1 and Table S2. Download spectrum.01410-22-s0001.pdf, PDF file, 0.2 MB [file spectrum.01410-22-s0001.pdf]

Gene content similarities of P1-like phage-plasmids and their implication in characterizing and spreading a CTX-M-55 resistant to 3rd cephalosporins

Mianzhi Wang[1,2,3]†, Li Jiang[1,2,3]†, Jingyi Wei[1,2,3], Heng Zhu[1,2,3], Junxuan Zhang[5,6,7], Ziyi Liu[1,2,3], Wenhui Zhang[1,2,3], Xiaolu He[5,6,7], Yuan Liu[1,2,3], Ruichao Li[1,2,3], Xia xiao[1,2,3], Yongxue Sun[5,6,7,8], Zhenling Zeng[5,6,7,8], Zhiqiang Wang[1,2,3,4]*

[1]College of Veterinary Medicine, Yangzhou University, Yangzhou, Jiangsu 225009, China

[2]Jiangsu Co-Innovation Center for Prevention and Control of Important Animal Infectious Diseases and Zoonoses, Yangzhou, 225009, China

[3]Priority Academic Program Development of Jiangsu Higher Education Institutions (PAPD), Yangzhou, 225009, China

[4]International Research Laboratory of Agriculture and Agri-Product Safety, the Ministry of Education of China, Yangzhou, Jiangsu 225009, China

[5]College of Veterinary Medicine, South China Agricultural University, Guangzhou 510642, China

[6]National Laboratory of Safety Evaluation (Environmental Assessment) of Veterinary Drugs, Guangzhou 510642, China

[7]National Risk Assessment Laboratory for Antimicrobial Resistance of Animal Original Bacteria, Guangzhou 510642, China

[8]Guangdong Laboratory for Lingnan Modern Agriculture, Guangzhou, China.

* Corresponding author:

Email: zqwang@yzu.edu.cn

† These authors contributed equally to this work.

Table S1:Information of 77 P1-like community

P_P_subgroup	NCBI_accession	NCBI_name	genome_size_bp	inc_type	repL
NA	NZ_CP016381	Aeromonas hydrophila strain AHNIH1 plasmid pASP-135	143348	ND	ND
NA	NZ_CP013486	Vibrio alginolyticus strain ATCC 33787 plasmid pMBL128	128112	ND	ND
NA	NZ_CP009882	Pantoea sp. PSNIH1 plasmid pPSP-26e	87166	ND	ND
NA	NZ_CP012732	Shigella flexneri 1a strain 0228 plasmid	25194	ND	ND
NA	NZ_CP024288	Escherichia albertii strain 2014C-4356 plasmid unnamed6	19118	ND	ND
NA	NZ_CP018459	Klebsiella pneumoniae strain Kp_Goe_39795 plasmid pKp_Goe_795-4	16971	ND	ND
NA	NZ_CP038627	Arsenophonus nasoniae strain FIN plasmid pArsFIN15	15977	ND	ND
P1_subgroup1	NZ_CP021203	Escherichia coli strain Z1002 plasmid p1002-1	183508	(2) IncFII;p0111	repL
P1_subgroup1	NZ_CP034821	Salmonella sp. SSDFZ54 plasmid pTB502	146389	(1) p0111	repL
P1_subgroup1	NZ_CP016549	Escherichia coli strain O177:H21 plasmid unnamed3	126046	(1) IncY	repL
P1_subgroup1	NZ_CP029117	Escherichia coli strain AR435 plasmid unnamed4.	120846	(1) IncY	repL
P1_subgroup1	NZ_CP019054	Escherichia coli strain CRE1540 plasmid p1540-3	116028	(1) p0111	repL
P1_subgroup1	NC_042128	Escherichia phage RCS47	115154	(1) p0111	repL
P1_subgroup1	NZ_LR130557	Escherichia coli strain MS14385 genome assembly	112419	(1) p0111	repL
P1_subgroup1	NZ_CP026475	Escherichia coli strain KBN10P04869 plasmid pKBN10P04869B	104701	(1) IncY	repL
P1_subgroup1	NC_031129	Salmonella phage SJ46	103445	(1) p0111	repL
P1_subgroup1	NZ_CP030784	Escherichia albertii strain 2012EL-1823B plasmid unnamed1	100347	(1) p0111	repL
P1_subgroup1	NZ_AP018809	Escherichia coli E2865 plasmid pE2865-1 DNA	99498	(1) p0111	repL
P1_subgroup1	NZ_CP028125	Escherichia coli O26 str. RM10386 plasmid pRM10386-1	98899	(1) p0111	repL
P1_subgroup1	NZ_CP011063	Escherichia coli str. Sanji plasmid pSJ_98	98436	(1) IncY	repL
P1_subgroup1	NZ_CP015837	Escherichia coli strain MS6198 plasmid pMS6198C	98242	(1) p0111	repL
P1_subgroup1	NZ_CP015997	Escherichia coli strain S51 plasmid pS51_2	98216	(1) p0111	repL
P1_subgroup1	NC_013370	Escherichia coli O111:H- str. 11128 plasmid pO111_2 DNA	97897	(1) p0111	repL
P1_subgroup1	NZ_CP013030	Escherichia coli strain 2012C-4227 plasmid unnamed2	97704	(1) p0111	repL
P1_subgroup1	NZ_CP021537	Escherichia coli strain AR_0119 plasmid unitig_3	97474	ND	ND
P1_subgroup1	NZ_LT905089	Salmonella enterica subsp. enterica serovar Typhi strain ty3-243 genome assembly	97394	(1) p0111	NZ_LT905089
P1_subgroup1	NZ_CP009051	Escherichia coli NCCP15648 plasmid p15648-1	97258	(1) IncY	repL

P1_subgroup1	NZ_CP027133	Escherichia coli strain AR_0372 plasmid unnamed4.	97167	(1) p0111	repL
P1_subgroup1	NZ_CP026725	Escherichia coli strain 266917_2 plasmid p266917_2_02	97124	(1) IncY	repL
P1_subgroup1	NZ_CP033848	Escherichia coli strain FDAARGOS_497 plasmid unnamed2	97018	(1) p0111	repL
P1_subgroup1	NZ_CP024817	Escherichia coli strain CREC-629 plasmid pCREC-629_2	96990	(1) IncY	repL
P1_subgroup1	NZ_CP024832	Escherichia coli strain CREC-532 plasmid pCREC-532_2	96987	(1) IncY	repL
P1_subgroup1	NZ_CP019075	Escherichia coli strain CRE1493 plasmid p1493-4	96986	(1) IncY	repL
P1_subgroup1	NZ_CP033882	Escherichia coli strain 50579417 plasmid p50579417_1	96948	(1) p0111	repL
P1_subgroup1	NZ_CP023961	Escherichia coli strain FDAARGOS_448 plasmid unnamed2	96807	(1) IncY	repL
P1_subgroup1	NZ_CP022733	Escherichia coli strain SA186 plasmid pSA186_4	96658	(1) p0111	repL
P1_subgroup1	NZ_CP035313	Escherichia coli strain D72 plasmid pD72-mcr1	96320	(1) p0111	repL
P1_subgroup1	NZ_CP034164	Escherichia albertii strain 2014C-4015 plasmid p2014C-4015-2	96264	(1) p0111	repL
P1_subgroup1	NZ_CP027575	Escherichia coli strain 2013C-4081 plasmid unnamed2.	95952	(1) p0111	repL
P1_subgroup1	NZ_CP027199	Escherichia coli strain WCHEC025943 plasmid p1_025943	95895	(1) IncY	repL
P1_subgroup1	NZ_CP012491	Escherichia coli strain CFSAN004176 plasmid pCFSAN004176P_03	95721	(1) IncY	repL
P1_subgroup1	NZ_CP034163	Escherichia albertii strain 06-3542 plasmid p06-3542	95683	(1) p0111	repL
P1_subgroup1	NZ_CP019188	Salmonella enterica subsp. enterica serovar Pomona str. ATCC 10729 plasmid pATCC10729_02	95279	(1) p0111	repL
P1_subgroup1	NZ_CP027443	Escherichia coli strain 2013C-3252 plasmid unnamed1	95157	(1) p0111	repL
P1_subgroup1	NZ_CP009168	Escherichia coli 1303 plasmid p1303_95	94959	(1) IncY	repL
P1_subgroup1	NC_005856	Enterobacteria phage P1	94800	(1) IncY	repL
P1_subgroup1	NZ_AP018798	Escherichia coli E2855 plasmid pE2855-2 DNA	94446	(1) p0111	repL
P1_subgroup1	NZ_CP010173	Escherichia coli strain H8 plasmid A	94395	(1) IncY	repL
P1_subgroup1	NZ_CP030183	Salmonella enterica strain SA20030575 plasmid pSA20030575.2	94179	(1) IncY	repL
P1_subgroup1	NC_017653	Escherichia coli O55:H7 str. RM12579 plasmid p12579_1	94015	(1) p0111	repL
P1_subgroup1	NZ_CP030188	Salmonella enterica strain SA20094620 plasmid pSA20094620.3	93719	(1) IncY	repL
P1_subgroup1	NZ_CP023732	Escherichia coli strain FORC 064 plasmid pFORC64.1	93276	(1) IncY	repL
P1_subgroup1	NZ_CP027590	Escherichia coli strain 2014C-3011 plasmid unnamed2.	92449	(1) p0111	repL
P1_subgroup1	NZ_CP038506	Escherichia coli strain 28Eco12 plasmid p28Eco12	92027	(1) IncY	repL
P1_subgroup1	NZ_CP023381	Escherichia coli strain 127 plasmid p91	91199	(1) IncY	repL
P1_subgroup1	NZ_CP034959	Escherichia coli strain WCHEC020032 plasmid p1_020032	90842	(1) IncY	repL

P1_subgroup1	NZ_CP029103	Escherichia coli strain AR437 plasmid unnamed1	89643	(1) IncY	repL
P1_subgroup1	NZ_CP031654	Escherichia coli strain UK_Dog_Liverpool plasmid pCARB35_01	89643	(1) IncY	repL
P1_subgroup1	NZ_CP021336	Escherichia coli strain 95JB1 plasmid p95JB1A	86922	(1) p0111	repL
P1_subgroup1	NZ_CP021340	Escherichia coli strain 95NR1 plasmid p95NR1A	86917	(1) p0111	repL
P1_subgroup1	NZ_CP023896	Escherichia coli strain FDAARGOS_433 plasmid unnamed3	83988	(1) IncY	repL
P1_subgroup1	NZ_CP027223	Escherichia coli strain 2015C-3101 plasmid unnamed2.	72543	(1) p0111	repL
P1_subgroup2	NZ_CP027309	Escherichia coli strain 2015C-3108 plasmid unnamed2	93724	ND	ND
P1_subgroup2	NZ_AP018804	Escherichia coli E2863 plasmid pE2863-2 DNA	92925	ND	ND
P1_subgroup2	NZ_CP032875	Escherichia coli strain WCHEC000837 plasmid p1_000837	92538	ND	ND
P1_subgroup2	NZ_CP028585	Escherichia coli strain WCHEC4533 plasmid p1_000533	92537	ND	ND
P1_subgroup2	NZ_CP022228	Escherichia coli strain WCHEC96200 plasmid p1_000200	92388	ND	ND
P1_subgroup2	NZ_CP028198	Salmonella enterica subsp. enterica serovar Concord strain CFSAN018747 plasmid pGMI14-002_2	92366	ND	ND
P1_subgroup2	NZ_CP034790	Escherichia coli strain ECCNB20-2 plasmid pTB423	91762	ND	ND
P1_subgroup2	NZ_CP020051	Escherichia coli strain AR_0118 plasmid unitig_3	91291	ND	ND
P1_subgroup2	NZ_CP029365	Escherichia coli strain WCHEC035148 plasmid p1_035148	89248	ND	ND
P1_subgroup2	NZ_CP011430	Salmonella enterica subsp. enterica strain YU39 plasmid pYU39_89	88949	ND	ND
P1_subgroup2	NZ_CP026818	Shigella dysenteriae strain 96-265 plasmid unnamed1	80213	ND	ND
P1_subgroup2	NZ_CP026808	Shigella dysenteriae strain 204/96 plasmid unnamed1	79695	ND	ND
P1_subgroup2	NZ_CP026821	Shigella dysenteriae strain 96-3162 plasmid unnamed1	79586	ND	ND
P1_subgroup2	NZ_CP026816	Shigella dysenteriae strain 93-119 plasmid unnamed1	79553	ND	ND
P1_subgroup2	NZ_CP027382	Escherichia coli strain 2013C-3250 plasmid unnamed2.	36491	ND	ND

ND: Not deteced

Table S2：Primers for the detection of P1-like PPs using nine specfic genes.

Genes	Primer sequence (5'-3')		AS[a]	MT[b]	Reference
16S rRNA (V3-V4)	F- CCTACGGGRSGCAGCAG	R- TACNVGGGTATCTAATCC	445	55	(1)
repL	F- GCCAATCAACCGTCGTTCGT	R- AAGCATATTTCCGCGCTGCC	451	54.5	(2)
gp22	F- GCCCAGACTACTTCCCATTTAT	R- CTCTGGAAGCCCGTTCTAAAT	919	53.6	In this study
gp23	F- GACTTGGGAACAGGGTGTATT	R- GCGCAGTTCGGTACTGATAA	784	52.4	In this study
gp25	F- GCCAGTTCGCCTTTGAAATAG	R- GCAGTCCACCAGCATTGATA	528	52.4	In this study
pmgS	F- GCTTCACGCACAGCTTATTG	R- GAAGAACTCTACCCAACCTTCC	802	54	In this study
pdcB	F- CCACGCTGAACGAGACAATA	R- CGTGCGTCTTCTCCCAATTA	638	51	In this study
ppp	F- CCGTATCGACGGTACCAAATAC	R- CCGGAGTGTGACCAAAGATAAA	551	54	In this study
hdf	F- GCGACTGAGCGTTAGGAATAG	R- CCCTTATCACGGCGATCAATAA	204	55	In this study
pacA	F- ACAGCCGCAAGCCAAATA	R- CGTTCTCCAGCATAAGGAGATG	414	53	In this study

PCR procedure was as follows: 95 °C denaturation for 3 min, followed by 40 cycles consisting of denaturation (95 °C for 15 s), annealing for 30 s (the melting temperature shown as above).

[a] Amplicon size (bp).

[b] Melting temperature (°C)

1. Klindworth A, Pruesse E, Schweer T, Peplies J, Quast C, Horn M, Glockner FO. 2013. Evaluation of general 16S ribosomal RNA gene PCR primers for classical and next-generation sequencing-based diversity studies. Nucleic Acids Res 41:e1.
2. Billard-Pomares T, Fouteau S, Jacquet ME, Roche D, Barbe V, Castellanos M, Bouet JY, Cruveiller S, Medigue C, Blanco J, Clermont O, Denamur E, Branger C. 2014. Characterization of a P1-like bacteriophage carrying an SHV-2 extended-spectrum beta-lactamase from an Escherichia coli strain. Antimicrob Agents Chemother 58:6550-7.
